# GD2-targeting therapy: a comparative analysis of approaches and promising directions

**DOI:** 10.3389/fimmu.2024.1371345

**Published:** 2024-03-15

**Authors:** Julia Philippova, Julia Shevchenko, Sergey Sennikov

**Affiliations:** Laboratory of Molecular Immunology, Federal State Budgetary Scientific Institution Research Institute of Fundamental and Clinical Immunology, Novosibirsk, Russia

**Keywords:** disialoganglioside GD2, cancer immunotherapy, monoclonal antibody, cell therapy, CAR cells, vaccine, neuroblastoma, clinical trials

## Abstract

Disialoganglioside GD2 is a promising target for immunotherapy with expression primarily restricted to neuroectodermal and epithelial tumor cells. Although its role in the maintenance and repair of neural tissue is well-established, its functions during normal organism development remain understudied. Meanwhile, studies have shown that GD2 plays an important role in tumorigenesis. Its functions include proliferation, invasion, motility, and metastasis, and its high expression and ability to transform the tumor microenvironment may be associated with a malignant phenotype. Structurally, GD2 is a glycosphingolipid that is stably expressed on the surface of tumor cells, making it a suitable candidate for targeting by antibodies or chimeric antigen receptors. Based on mouse monoclonal antibodies, chimeric and humanized antibodies and their combinations with cytokines, toxins, drugs, radionuclides, nanoparticles as well as chimeric antigen receptor have been developed. Furthermore, vaccines and photoimmunotherapy are being used to treat GD2-positive tumors, and GD2 aptamers can be used for targeting. In the field of cell therapy, allogeneic immunocompetent cells are also being utilized to enhance GD2 therapy. Efforts are currently being made to optimize the chimeric antigen receptor by modifying its design or by transducing not only αβ T cells, but also γδ T cells, NK cells, NKT cells, and macrophages. In addition, immunotherapy can combine both diagnostic and therapeutic methods, allowing for early detection of disease and minimal residual disease. This review discusses each immunotherapy method and strategy, its advantages and disadvantages, and highlights future directions for GD2 therapy.

## 1 Introduction

Tumor immunotherapy targeting tumor-associated antigen (TAA) using monoclonal antibodies (mAbs) or immunocompetent cells can improve standard therapeutic methods, including surgery, chemotherapy, and radiation. The immunologic approach aims to stimulate and train the body’s own immune system to cope with malignant cells, which is a safer approach (1). In addition, immunotherapy can combine both diagnostic and therapeutic methods, allowing for early detection of the disease and minimal residual disease. Immunotherapy is also an effective method for combating metastasis and chemoresistant diseases, and treatment with mAbs shows encouraging results in long-term and overall relapse-free survival (2, 3).

Disialoganglioside GD2 is a surface TAA that is expressed by a wide range of tumors of neuroectodermal and epithelial origin, such as neuroblastoma (4), melanoma (5), glioma (6), retinoblastoma (7), medulloblastoma (8), small-cell lung cancer (9), various types of breast cancer (10, 11) and sarcoma (12–14) bladder cancer (15), colorectal cancer (16), and prostate cancer (17). GD2 can be detected on normal central and peripheral nervous system cells, melanocytes (18), lymphocytes, dendritic cells (19), and mesenchymal stem cells (20). Nevertheless, GD2 expression is significantly higher in tumor cells, making this target suitable not only for therapy but also for diagnosis and assessment of disease prognosis (21). GD2 also possesses genetic stability, i.e., the expression level does not decrease during treatment, and most of the antigen remains on the cell surface after binding by antibodies and recognition by immune cells (22). At the same time, GD2 immunotherapy has some limitations, mainly related to the occurrence of side effects and low efficacy in the treatment of extensive solid masses. In this review, different approaches to GD2 immunotherapy, their advantages and disadvantages, and the search for new strategies to improve current developments are presented.

## 2 Structure and synthesis of disialoganglioside GD2, its role and function

### 2.1 Structure and synthesis of disialoganglioside GD2

Ganglioside GD2 is a carbohydrate-containing sphingolipid (glycosphingolipid) consisting of a ceramide (sphingosine linked by an amide group to a fatty acid) with two sialic acid residues attached via three monosaccharide links (23, 24). The intracellular synthesis of GD2 occurs in the Golgi apparatus, which starts with the formation of ceramide (lipid domain) (25), followed by the addition of monosaccharide links by means of glycosyltransferases – GM3 synthetase (ST3Gal V) and GD3 synthetase (ST8Sia I, GD3S) (23, 25). The lipid domain is then incorporated into the plasma membrane, whereas the carbohydrate residues are located on the cell surface. GD2 is synthesized from the ganglioside precursors GD3 or GM3 by β1,4-N-acetylgalactosaminyltransferase (GalNAcT, GD2S).

### 2.2 Function of disialoganglioside GD2 and its role in oncogenesis

The functions of GD2 during normal development of the organism are understudied; it is assigned a role in the maintenance and repair of neural tissue through the regulation of complement activation and inflammation (26). At the same time, numerous studies demonstrate the importance of GD2 in oncogenesis; and its function, high expression, and ability to exert remodeling effects on the tumor microenvironment (TME) may be associated with malignant phenotypes. GD2 can promote proliferation, invasion, motility, and metastasis of various tumor cell types (27, 28), by inducing phosphorylation through the hepatocyte growth factor (HGF) receptor and c-Met pathway of breast cancer (29) or tyrosine kinase receptors and FAK pathways of osteosarcoma (30). ASC amino acid transporter 2 (ASCT2) promotes the malignant phenotype of small-cell lung cancer by enhancing cellular uptake of glutamine, leading to enhanced cell proliferation and migration through phosphorylation of the mTOR1 pathway (31). GD2 also plays a key role in melanoma cell adhesion, growth, proliferation, and invasion by interacting with integrin β1 (32). The ST8SIA1 (GD3 synthetase) gene was shown to regulate GD2 biosynthesis; and when it is knocked out, the inhibition of the FAK/AKT/mTOR signaling pathway and suppression of growth and metastasis in breast cancer is observed (33). It was also reported that increased GD2 expression in cancer cells is associated with NF-κB, and treatment with IKK (inhibition of NF-κB signaling) inhibitors in an experimental model reducing breast cancer metastasis to the lung by more than 5-fold (34), which also suggests the influence of GD2 on metastasis and cell migration. In addition, high GD2 expression is characteristic of diffuse mediastinal glioma cells with the H3K27M mutation, a rare but quite aggressive malignancy (35). GD2 expression was shown to be elevated in oral malignant osteosarcoma samples (30) and neuroblastoma with MYCN amplification (36), which also negatively affects the forecast. Recently, sialic acid-binding Ig-like lectins Siglec-7 were discovered to be expressed on NK cells (37). GD2 is able to suppress NK cell function through binding to Siglec-7, thereby maintaining immunosuppressive TME (38). In addition, GD2 also inhibits the functional activity of T cells and dendritic cells (39), while promoting the recruitment of MDSCs (myeloid-derived suppressor cells) (40) and Tregs (regulatory T cells) (41) to TME. Anti-GD2 mAb treatment inhibits the mTOR/MAPK signaling pathway in breast cancer cells (42), which results in inhibition of tumor migration and growth, and competes with Siglec-7 for binding to GD2 (38).

## 3 Monoclonal antibodies

**Figure 1:**
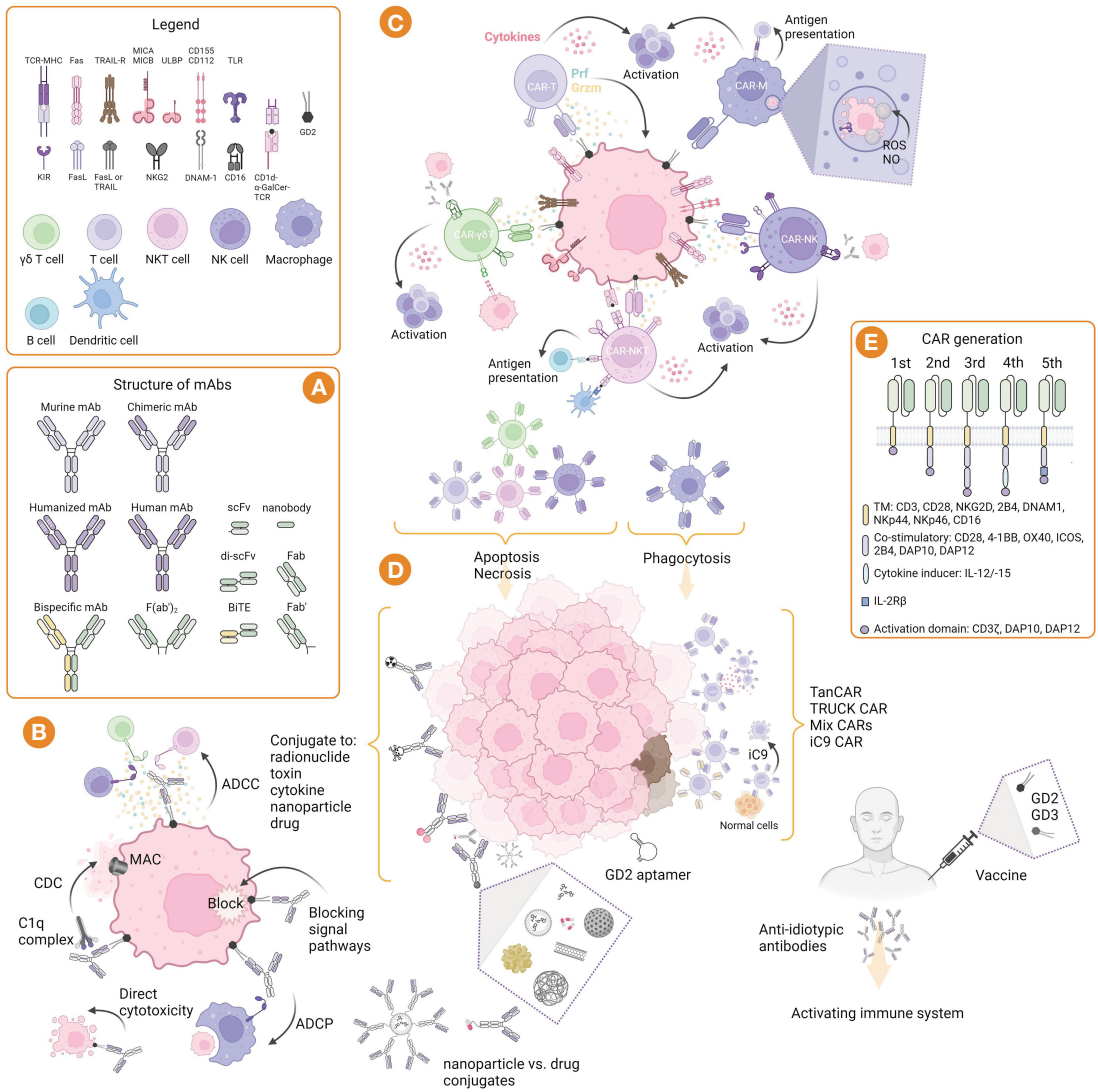
GD2-targeted immunotherapy: strategies, structure, and mechanisms of action. **(A)** Structure of murine, chimeric, humanized and bispecific mAbs, and their derivatives such as nanobodies and bispecific T cell engager (BiTE). **(B)** Mechanism of mAbs action: induction of CDC involving complement component 1q complex, followed by the complement cascade and formation of the membrane attack complex (MAC); induction of ADCC mediated by γδ T cells, NK cells, and NKT cells, as well as ADCP mediated by macrophages; blocking of signal pathways and direct cytotoxicity by induction of apoptosis. **(C)** Mechanisms of immune effector cell cytotoxicity that allow their properties to be exploited in adoptive and CAR therapies. **(D)** Strategies of immunotherapy include nacked mAbs, as well as conjugated mAbs with radionuclides, toxins, cytokines, nanoparticles, and drugs; CAR cells can be used alone or directed to two or more targets, as well as their modifications, such as TanCAR (bispecific CAR), TRUCK (T cells redirected for antigen-unrestricted cytokine-initiated killing) CAR, and iC9 (inducible caspase 9) CAR; GD2 aptamer can be conjugated to other molecules and toxins for drug delivery or imaging; GD2 vaccines can be used alone or in combination with GD3 vaccines to form anti-idiotypic antibodies and activate the immune system. **(E)** Structure of CARs generation including domains for αβ and γδ T cells, NK cells, NKT cells, and macrophages. Created with BioRender.com.

Since the 1980s, anti-GD2 mAbs have been actively investigated as theranostic agents in cancer immunotherapy. The unconjugated antibodies recognize TAAs and bind to surface receptors of tumor and immunocompetent TME cells, and depending on the type of the receptor, exert antitumor effects through various mechanisms, including antibody-dependent cell-mediated cytotoxicity/antibody-dependent cellular phagocytosis (ADCC/ADCP), complement-dependent cytotoxicity (CDC), and direct cytotoxicity (**Figure 1**). During ADCC/ADCP, mAbs bind to Fcγ receptors and promote destruction (NK cells (43, 44), neutrophils (45), γδ T-cells (46)) or phagocytosis (macrophages (47)) of tumor cells. In CDC, the classical complement pathway is activated with the formation of the membrane attack complex (MAC) and recruitment of NK-, T-, NKT-cells, neutrophils, macrophages, and dendritic cells (48). Direct cytotoxicity is realized by the blockade of growth factor receptors, with mAbs binding to receptors on the membrane surface or soluble forms, or inducing apoptosis or necrosis axes (49). mAbs against GD2 can exert a direct cytotoxic action on gangliosides, likely leading to mitochondrial damage by translocation of GD2 from the cell membrane to intracellular compartments (50).

Three anti-GD2 drugs dinutuximab (Unituxin^®^), dinutuximab beta (Qarziba^®^), and naxitamab (Danyelza^®^) were formally approved in clinical practice for the treatment of patients with high-risk neuroblastoma. Despite clinical successes, there are several therapy-limiting challenges, including sensitization-related side effects, immunosuppressive TME, loss of antigen expression, production of neutralizing human anti-murine/-chimeric/-human antibodies (HAMA, HACA, and HAHA), extensive masses, etc. Therefore, new strategies are required to modify antibodies and conjugate/combine with other drugs for successful treatment (**Table 1**). **Table 2** shows comparative characteristics of murine, chimeric, and humanized mAbs.

**Table 1:** Approaches to anti-GD2 therapies: features, problems, and strategies.

	Features	Problems	Strategies		
mAbs	Clinical use is widespread Effective ling-term antitumor efficacy Increase overall survival Approved drugs Less toxic than chemotherapy drugs	Adverse effects Blood–brain barrier (BBB) Rapid half-life TME	Humanized mAbs Removal of IL-2 from the standard regimen IgA-based antibody/ch14.18 with H3-16 IgG1m4 mutation Locoregional delivery Continuous flow long infusions Modified glycosylation profile and humanization Gene transfer technology and *in vivo*/ex vivo antibody production Combination with GM-CSF, ICI, anti-CD47 (magrolimab), TGFβR1 inhibitor, isotretinoin, and chemotherapy Allogeneic transfer of NK or T cells	
CAR T cells	Pass through BBB Persistence duration Possess potent cytotoxic activity Increased antigen-binding capacity is due to avidity and polyvalence Bind to cell with lower levels of TAA	Exhaustion and decreased proliferative capacity Non-tumor toxicity TME	Improving manufacturing protocols: produce T cells with less exhaustion and phenotypes of naive and central memory cells, decrease culture time Non-virial transduction: piggyBac or CRISPR/Cas9 Decreased tonic signaling CAR T with iCasp9 Locoregional delivery Armored CAR-T secreting cytokines (IL-7/-12/-18/-23), IL-7R or chemokines (IL7, CCR2b) TanCAR GITRL, PD-1, or BiTE-expressing CAR-T Combination with ICI (nivolumab pembrolizumab), anti-VEGF (bevacizumab), IGF1R/IR inhibitor (linsitinib), BRAF inhibitors (dabrafenib, vemurafenib) and MEK inhibitors (trametinib, cobimetinib), oncolytic viruses, trans-retinoic acid (ATRA), and chemotherapy	
Immunocytokines (IC)	Targeted delivery of cytokines	Large molecule size Low tumor penetration through blood vessels	Fusion proteins (RLI) Locoregional delivery IL-15/-21 or GM-CSF based IC for TME remodeling	
Immunotoxins	Antitumor properties of toxins	Immunogenicity	Modification of toxin structure, deimmunization Humanized mAbs	
Radiolabeled mAbs	Theranostic therapy Radionuclide enhances antitumor activity	Toxicity	Multi-step infusion with bispecific mAbs Less toxic radiotracer and humanized mAbs	
Drug conjugated mAbs	Drug delivery to tumor site Less toxic than IC	Low tumor stability and accumulation	Linker modification Nanobody fragments, but this disables ADCC and CDC	
Nanoparticles conjugated mAbs	Improved drug delivery to tumor site compared to drug conjugated mAbs Theranostic and photothermal therapies Properties depend on material: organic and non-organic materials	Low tumor stability and accumulation, toxicity	Modification of nanoparticle size, shape and surface charge Biodegradable and biocompatible polymers Fragments of mAbs and less toxic particles GD2 aptamer	
Bispecific mAbs	Recognition of TAA and recruitment of cytotoxic cells Trifunctional mAbs additionally attract APCs	Rapid half-life	Optimization of structure and spatial configuration Increased molecular weight, tetravalent antibodies and metal complexes Continuous flow long infusions	
Vaccine	Modulating the immune system with minimal adverse effects	Low antitumor efficacy	Application of vaccines as an adjuvant therapy Bivalent vaccines with β-glucan	
Other GD2-targeting therapy: current clinical trials					
Vaccine	(recruiting) NCT04936529 NCT06057948 NCT00911560 (active, not recruiting)	Bivalent vaccine with adjuvant OPT-821 (QS-21) plus β-glucan and with/without GM-CSF Bivalent vaccine with adjuvant OPT-821 (QS-21) with/without β-glucan Bivalent vaccine with adjuvant OPT-821 (GD2L and GD3L) linked to KLH plus β-glucan	Neuroblastoma Neuroblastoma Neuroblastoma	Phase II Phase II Phase I/II

Current clinical trials of other anti-GD2 therapies.

**Table 2:** Comparison of murine, chimeric, and humanized mAbs. Current clinical trials of anti-GD2 mAbs.

	Murine	Chimeric	Humanized			
Structure	3F8 and 14.18 from murine IgG3 14G2a: IgG2a-class switch variant from 14.18 ME36.1: IgG2a- and IgG1-switch variant from murine IgG3	ch14.18: fusing heavy and light chains of 14.18 Dinutuximab generated by SP2.0 cells Dinutuximab β generated by CHO cells	hu14.18K322A: single amino acid substitution in the Fc region of K322A (humanized dinutuximab) generated by YB2.0 cells hu3F8: fusion of complementarity-determining regions with the human IgG1 framework (naxitamab)		
Binding affinity to the GD2 target	3F8 has higher affinity than ME36.1 and 14.G2a	ch14.18 and 14.G2a exhibit equal affinity	hu3F8 has a 10-fold higher affinity than ch14.18		
	m3F8 > hu3F8 > ch14.18					
ADCC CDC	14.G2a has higher ADCC than 14.18 3F8 has higher CDC than ch14.18	ch14.18 and 14.G2a are equally capable of mediation of CDC ch14.18 has a 50-100 fold higher ADCC than 14.G2a Dinutuximab β has higher ADCC than dinutuximab	mAbs generated by YB2/0 cells have higher ADCC than mAbs generated by CHO cells K322A mutation led to decreased CDC hu3F8 has higher ADCC (not CDC) than ch14.18		
	hu3F8 > ch14.18 > m3F8 m3F8 > ch14.18 > hu3F8					
Features and therapy	ME36.1: cross reaction with GD2 and GD3 3F8 and 14.G2a widely used as monotherapy	Less immunogenic than murine mAbs ch14.18 has a longer half-life than hu3F8 2 drugs officially approved Long-term results comparable to oral chemotherapy	Less immunogenic than chimeric mAbs hu14.18K322A was developed to reduce neuropathic toxicity and pain hu3F8 has significant antitumor efficacy Naxitamab officially approved		
Limitations and adverse effects	HAMA Most common adverse effects include allodynia, pain, hypertension, hypotension, apnea, tachycardia, fever, allergic reaction Treatment with 14G2a caused severe pain, with 3F8 caused reversible encephalopathy syndrome Less common adverse effects include hyponatremia/kalemia, nausea, vomiting, diarrhea, liver dysfunction, hypoxia	HACA Adverse effects comparable to murine mAbs Dinutuximab/beta treatment resulted in demyelinating polyneuropathy, and ocular signs present with ophthalmoplegia, mydriasis, and accommodation deficit Continuous infusion can only reduce pain intensity	HAHA hu14.18K322A has a higher HAHA response rate compared to hu3F8 Moderate adverse effects Treatment can be carried out on an outpatient basis		
	Treatment mAbs with IL-2 associated with capillary leak syndrome					
GD2-targeting therapy: current clinical trials with mAbs						
hu3F8	(active, not recruiting) NCT02650648 NCT01757626 (recruiting) NCT05489887 NCT06026657 NCT02502786 NCT03363373	hu3F8 plus NK cells, cyclophosphamide hu3F8 plus GM-CSF hu3F8 with/without ceritinib TGFβi NK cells plus gemcitabine with/without hu3F8 hu3F8 plus GM-CSF hu3F8 plus GM-CSF		Neuroblastoma Neuroblastoma Neuroblastoma Breast Cancers Osteosarcoma Neuroblastoma		Phase I Phase I/II Phase II Phase Ib/II Phase II Phase II
hu14.18K322A	NCT01857934 (active, not recruiting)	hu14.18K322A with induction chemotherapy		Neuroblastoma		Phase II
ch14.18/SP2.0	(active, not recruiting) NCT03786783 NCT01711554 (recruiting) NCT05400603 NCT03794349 NCT05421897	ch14.18/SP2.0 plus GM-CSF with chemotherapy ch14.18/SP2.0 plus lenalidomide with/without isotretinoin γδ T cells with ch14.18/SP2.0, temozolomide, irinotecan and zoledronate ch14.18/SP2.0, irinotecan and temozolomide and with/without eflornithine ch14.18/SP2.0 with chemotherapy		Neuroblastoma Neuroblastoma Neuroblastoma Neuroblastoma Neuroblastoma		Phase II Phase I Phase I Phase II Phase IV
ch14.18/CHO	(active, not recruiting) NCT02743429 (recruiting) NCT02914405 NCT05272371 NCT06071897 NCT05080790 NCT05754684 NCT01704716	ch14.18/CHO continuous infusion 131-1 mIBG followed by nivolumab and ch14.18/CHO ch14.18/CHO with chemotherapy ch14.18/CHO with induction chemotherapy ch14.18/CHO with zoledronic acid and IL-2 ch14.18/CHO plus NK cells, IL-2, GM-CSF and spironolactone ch14.18/CHO with induction chemotherapy plus isotretinoin with/without IL-2		Neuroblastoma Neuroblastoma Neuroblastoma Neuroblastoma and Ganglioneuroblastoma Leiomyosarcoma Neuroblastoma Neuroblastoma		Phase II Phase I Phase I Phase III Phase II Phase II Phase III

Antibody-dependent cell-mediated cytotoxicity/antibody-dependent cellular phagocytosis (ADCC/ADCP), complement-dependent cytotoxicity (CDC), human anti-murine/-chimeric/-human antibodies (HAMA, HACA, and HAHA), chinese hamster ovary (CHO), granulocyte-macrophage colony-stimulating factor (GM-CSF), transforming growth factor β imprinted (TGFβi) NK cells, metaiodbenzylguanidine (mIBG).

### 3.1 Murine mAbs: 3F8, 14G2a and ME36.1

Hybridoma technology was used to develop the first murine mAbs 3F8 and 14.18 of the IgG3 subclass (51, 52). Mouse mAb showed not only stable binding to GD2 antigen (53), but also the ability to mediate CDC (51) and ADCC (52, 54). Later, mAb 14G2a was developed based on the IgG2a-class switch variant of 14.18, which showed higher ADCC than 14.18 *in vitro* and *in vivo* (55). mAb ME36.1, derived from murine IgG3 and being IgG2a- and IgG1-class switch variants, can cross-link to GD2 and GD3 (56). In clinical practice, 3F8 (57–59) and 14G2a (60–62) were widely used as monotherapy. However, a high level of HAMA and several side effects were reported among patients. In particular, in a rat model, the development of severe pain requiring high doses of morphine was observed after the administration of 14G2a (63). In order to enhance the therapeutic potential of mAbs, including overcoming prolonged severe lymphopenia (64), GM-CSF (64–66), isotretinoin (13-cis-retinoic acid, a vitamin A derivative) (67), oral β-glucan (68, 69), and adoptive transfer of NK cells were added to 3F8 (70). mAb 14G2a was also tested in combination with IL2 (71). The results reported difficulty in treating bulky masses or progressive disease (64, 65) and the development of severe side effects (71). In addition, the development of posterior reversible encephalopathy syndrome (PRES) was observed with 3F8 treatment (72), which calls into question further testing of murine mAb.

### 3.2 Chimeric mAbs: ch14.18/SP2.0 (dinutuximab)

In order to reduce immunogenicity and neutralizing antibody levels, chimeric murine-human ch14.18 antibodies were developed by combining murine IgG3 mAb 14.18 (IgG2a switch variant 14G2a) chimeric fragments with Fc fragments of human IgG1 produced by the SP2.0 cell line (73). It was shown that ch14.18/SP2.0 and 14.G2a equally exhibited antitumor activity, antigen affinity, and ability to mediate CDC. However, ch14.18-mediated ADCC *in vitro* was 50-100-fold more effective compared to 14.G2a (74). Pharmacokinetic analysis showed that ch14.18/SP2.0 had a longer half-life compared to 14G2a (75), but its clearance was accelerated after repeated administration, probably, due to HACA formation (76). At the same time, the CDC is higher in mouse antibody 3F8 than in ch14.18, which is due to the difference between human and mouse IgG1 and IgG3 immunoglobulins (77).

Studies of monotherapy with dinutuximab (76, 78–81) did not show any treatment benefit except for reduced immunogenicity. However, in the long term, the antitumor effect was comparable to the use of oral chemotherapy (82). Combination therapy of dinutuximab with IL-2 and/or GM-CSF was also evaluated in several studies (83–85), and in combination with the murine antibody R24 (86). Administration of cytokines enhances ADCC (83, 84, 86), but HACA titers get increased in response to chimeric antibody administration (85). The Children’s Oncology Group reported improved survival with the combination of dinutuximab with GM-CSF, IL-2, and isotretinoin (ANBL0032) (87) compared to standard therapy with isotretinoin (88), prompting the FDA and EMA to approve this combination for maintenance therapy of high-risk neuroblastoma in pediatric patients after ASCT (89). Subsequent ANBL0032 studies of the same patient cohort questioned the use of IL2 as a therapeutic agent, as no benefit was found and GM-CSF may induce an endogenous IL2 response (90).

Although immunotherapy with mAb showed encouraging results, the problem of delayed relapses remains relevant and requires the development of new methods and drugs. One approach may be aimed at modulating TME. Thus, it was shown that the addition of irinotecan and temozolomide chemopreparations to dinutuximab with GM-CSF would enhance the antitumor effect at minimal doses of mAb (ANBL1221) (91). It was also shown that γδ T cells can provide better antitumor activity in combination with dinutuximab and temozolomide, while being superior to αβ T cells due to their functional properties (92). Magrolimab (anti-CD47 mAb) (38), galunisertib (TGFβR1 inhibitor) (93), and anti-CD105 (94) may be added to dinutuximab to enhance its efficacy. The addition of magrolimab can provide potent synergism with dinutuximab and enhance the antitumor response toward phagocytosis, while anti-CD105 induces ADCC by cells expressing the Fc receptor. In mouse models, it was shown that immunotherapy with dinutuximab in combination with NK cells, initiated prior to tumor resection, can reduce disease severity and increase survival (95). Another approach is to modify mAb and improve the delivery method. Silk fibroin was proposed as a delivery platform for bioactive dinutuximab, which can provide a higher concentration of mAb in the tumor (96).

### 3.3 Chimeric mAb: ch14.18/CHO (dinutuximab beta)

The technology to produce ch14.18 according to GMP standards was based on antibody production by SP2.0 and NS0 cell lines, which are non-secreting murine melanoma cells that carry murine xenotropic retrovirus, making it much more difficult to purify antibodies for the use in clinical trials (97). The antibodies produced by the chinese hamster ovary (CHO) cell line are similar in structure to human serum antibodies and have a glycosylation type involving small amounts of sialic N-glycolylneuroamic acid, which provides a prolonged half-life and a reduced immunogenicity profile (98). In addition, CHO does not carry murine retrovirus, so in order to improve production, the CHO cell line was used to produce dinutuximab beta (ch14.18/CHO). Comparative analysis of ch14.18/CHO and ch14.18/SP2.0 showed similar CDC for the antibodies *in vitro*, while ADCC was higher for dinutuximab beta even at low antibody concentrations. *In vivo* evaluation revealed suppression of metastasis in the animal model, which was probably due to the enhancement of NK-depended ADCC (99). The SIOPEN (The International Society of Pediatric Oncology Europe Neuroblastoma group) clinical trial confirmed the feasibility of dinutuximab beta because the toxicity and pharmacokinetics profile were similar to dinutuximab with objective responses (100). Subsequent SIOPEN clinical trials of a combination of dinutuximab beta with/without subcutaneous administration of IL2, isotretinoin, and standard chemotherapy regimens showed improved 5-year survival. However, due to side effects and lack of benefit, IL2 is not recommended for further use (101–103).

The use of different regimens and combinations of dinutuximab beta with different therapeutic approaches was also actively explored in recent studies. The clinical use of dinutuximab beta and haploidentical stem cell transplantation (haplo SCT) can improve survival with an acceptable toxicity profile (104) and a low risk of graft versus host reaction (GvHD) induction (105). In addition, dinutuximab beta stimulates haplo SCT towards NK cell differentiation with enhanced ADCC and potent secretion of pro-inflammatory cytokines (sIL2R, TNFα, and IL6), which emphasizes combinational functionality (106). Application of immunocytokine FAP-IL-2v related to fibroblast activation protein stimulates NK-mediated ADCC without induction of Treg compared to IL2 (107). The addition of γδ T cells and dinutuximab beta also promotes ADCC-mediated tumor cell lysis, and systemic administration of zoledronic acid is safe and leads to T cell expansion (108). Prolonged infusion (109) or the use of dinutuximab beta immediately after induction therapy (110) demonstrate an acceptable toxicity profile and objective responses. In particular, the use of at least one cycle of dinutuximab beta before surgery can lead not only to remission but also to tumor necrosis and normalization of oncomarkers (110). In addition, prolonged infusion not only results in effective immunomodulation, but also allows for reduced pain toxicity (111). It was also shown that dinutuximab beta, despite its antitumor activity, leads to MDSC induction (112). Therefore, the addition of chemical agents such as 5-FU or vorinostat (113), can suppress MDSC differentiation (112), and the use of nivolumab (a PD-1 inhibitor) eliminates their immunosuppressive effects (114). Clinical use of dinutuximab beta and nivolumab in two patients with relapsed/refractory neuroblastoma resulted in complete and good partial remission (115). The combination of dinutuximab beta with dual blockade of immune checkpoints PD-1 and TIGIT more effectively inhibits tumor growth compared to a single blocker (116).

### 3.4 Humanized mAbsAbs: hu14.18K322A and hu3F8 (naxitamab)

Antibody humanization involves optimization of the antibody variant region, which subsequently affects the frequency of immune response (HAMA and HACA) to the murine fragment, in particular, the elevation of complement component C3a and activation of the cascade (76). MAb hu14.18K322A has identical C-regions of IgG1-κ as ch14.18, except for a point mutation of the amino acid sequence replacing alanine with lysine 322 in the C(H)2 domains of the Fc fragment (117), which prevents complement activation (118). In addition, antibodies produced by the cell line of the rat hybridoma YB2/0 cell line strongly mediate ADCC as a result of reduced fucosylation compared to CHO-derived antibodies (119), which was confirmed in preclinical studies (120). However, hu14.18K322A has a reduced ability to mediate CDC and is less likely to induce mechanical allodynia in animal models compared to dinutuximab (120). Retrospective analysis also confirmed a difference in pain side effects between hu14.18K322A and dinutuximab, with the use of humanized antibodies requiring less opioids (121). Preclinical studies showed that hu14.18K322A in combination with αCD40/CpG enhanced NK-dependent antitumor response (122), and was also nonspecifically taken up by tumor cells (123). The combination of hu14.18K322A, IL15Rα/IL15, and GM-CSF was also shown to induce greater tumor regression *in vivo* compared to therapy with hu14.18K322A and GM-CSF with/without IL2 (124). Clinical studies showed that HAMA production was observed in 40% of patients (125), and the concentration of hu14.18K322A required for cell lysis was 3.5-4 times lower than that of dinutuximab (126). The combination of hu14.18K322A with NK cells, cytokines, or chemopreventive agents can lead to clinically significant responses (126). The addition of hu14.18K322A to induction therapy resulted in an early antitumor response (127), and subsequent efficacy evaluation showed significant tumor shrinkage and an encouraging 3-year survival rate (128). A study of the pharmacokinetic profile showed no differences between daily and weekly regimens (129), demonstrating the advantage of hu14.18K322A over the long-term administration of dinutuximab.

Mouse antibody 3F8 was also humanized by transferring the complementarity determining region (CDR) of heavy and light chains to the human IgG1-κ framework based on their homology (130). It was shown that hu3F8 was 200-fold more effective in enhancing ADCC *in vitro* but mediated CDC less compared to m3F8, and was superior to other antibodies in its ability to bind to GD2 antigen and antitumor activity *in vivo*. Clinical use of hu3F8 in combination with GM-CSF revealed clear advantages in achieving significant antitumor results, durable response and safety (131), which prompted the FDA to formally approve naxitamab for the treatment of high-risk neuroblastoma (132). The HAMA response rate for hu3F8 was comparatively lower than for hu14.18K322A (131). Thus, naxitamab had low immunogenicity and required several cycles of treatment to provide comparable efficacy (133). The safety profile allows naxitamab to be used in an outpatient setting compared to dinutuximab, which requires an inpatient regimen (2, 3). The clinical benefit and long-term survival prospectively raise the question of replacing chemotherapy with autologous stem cell transplantation with naxitamab in combination with GM-CSF in patients with first complete remission (1). The advantages of utilization and distinctive properties over other mAbs make hu3F8 promising for use in various GD2 therapy strategies, including CAR-T cells and conjugated antibodies. It was also reported on the improved *in silico* affinity of hu3F8 with a single D32H mutation in CDR1-VL by altering the electrostatic surface potential, which enhanced *in vitro* and *in vivo* cytotoxicity while maintaining tissue specificity (134).

### 3.5 Anti-GD2 mAbs and neurotoxicity

Dose-limiting neurotoxicity induced by mAbs, which requires patient care and analgesic therapy, is one of the key issues to be addressed. Severe pain is believed to be caused by the binding of mAb to GD2 on nerve fibers (135), which locally activates CDC through the C1q binding domain, generating anaphylatoxins such as C5 or C3 (136). Hence, most studies have focused on reducing complement activation. Various approaches have been taken to modify monoclonal antibodies. Therefore, a modified version of murine 3F8 called heat-modified murine 3F8 (HM3F8) was created (137). This modified version lacks effector functions, specifically ADCC and CDC, and can target GD2 or cross-reactive epitopes on nerves, resulting in the prevention of neuropathic pain after subsequent administration of unmodified antibodies. A novel IgA-based version of ch14.18 has been developed to reduce neuropathic pain (138). Unlike the IgG-based version, IgA-based ch14.18 does not cause neurotoxicity due to the absence of a C1q binding site. A new form of ch14.18, derived from the IgA2 isotype and based on IgA3.0, has an extended elimination period, high stability, and does not cause neurotoxicity (139). A comparative analysis showed that humanized mAbs have lower CDC compared to mouse mAbs. As described above, hu14.18K322A, with a point mutation in the Fc-fragment of the C1q domain (117) was designed to reduce CDC and, therefore, neurotoxicity. However, a recent study showed that the K322A mutation has inconsistent complement activity and may not be effective for therapeutic purposes (140). Kulanthaivadivel et al. also suggested that FcγR-dependent cytotoxicity may cause neurotoxicity. Therefore, a proposed alternative mutation format for IgG2a does not bind to FcγR and C1q. It has also been reported that a humanized H3-16 IgG1m4 antibody with an Fc mutation based on ch14.18 can reduce CDC (141). In a rat pain model, H3-16 IgG1m4 demonstrated decreased allodynia compared to dinutuximab. Naxitamab is a potential candidate for outpatient use among the presented antibodies, but its therapy can be complicated by painful side effects. Therefore, reducing neurotoxicity remains an important issue.

O-acetyl-GD2 (OAcGD2) is a derivative of GD2 that is expressed by cancer tissues but not by peripheral nerves (142). This property allows to avoid neurotoxicity. Preclinical studies have shown that the murine antibody 8B6 targeting OAcGD2 inhibits tumor growth even in the absence of ADCC and CDC (143), and its chimeric form does not cause allodynic pain (144). Additional studies are required to evaluate the benefits of using antibodies that target OAcGD2 in reducing neurotoxicity compared to anti-GD2 antibodies.

### 3.6 Immunocytokines

Immunocytokines (ICs) were developed in order to provide targeted delivery directly to the target, and thus, achieve high concentrations in the TME and reduce systemic side effects. The first anti-GD2 IC was obtained by fusing the C-terminal CH3 domain of mAb ch14.18 to IL2, which showed more efficient antigen-binding activity compared to mAb (145). Preclinical studies showed that ch14.18-IL2 exerted commensurate activity with systemic administration of the cytokine (146) and provided a prolonged effect of IL2 by increasing the half-life (147). The ability of ch14.18-IL2 to induce T cells directly into the TME (148, 149), induce NK-depended ADCC and exert more effective antitumor activity compared to ch14.18 and/or IL2 (150, 151) was tested in animal models.

To reduce immunogenicity, hu14.18-IL2 was developed, which demonstrated similar antitumor mechanisms *in vivo* (152–154). Clinical use of hu14.18-IL2 showed activation/modulation of the immune system by increasing lymphocyte counts or sIL2R levels. However, no clinically significant effect was achieved against massive disease (155–159). Probable reasons for the low antitumor efficacy may be the large size of the IC molecule, which degrades as it passes through the liver (160) or has low permeability into the tumor from the bloodstream (161). Intratumoral administration of IC can provide a more effective antitumor effect than intravenous administration (162), and enhance migration of NK cells into the tumor focus (163). To reduce IL2-dependent side effects, IC was produced by fusing IL2 to the C-terminal of mAb hu14.18 light chains (164). This construct is thought to impede the binding of IL2 to IL2Rs of intermediate affinity, which are associated with the manifestation of side effects, allowing the targeting of high-affinity receptors responsible for antitumor effects. Separately, ICs based on IL15 and IL21, similar in structure and function to IL2, were developed, that were safer and capable of exerting a remodeling effect on TME (165). A study of ICs hu14.18-IL2/IL15/IL21 in combination with chemotherapy showed that hu14.18-IL15 and hu14.18-IL21 could induce complete tumor regression and improved survival compared with hu14.18-IL2, and their application contributed to an increase in CD8+ T cells and M1 and a decrease in Treg and MSDC in the tumor (166). IC based on hu14.18 and GM-CSF may serve as an alternative, with hu14.18-GM-CSF showing enhanced ADCC *in vitro* compared to hu14.18 and/or GM-CSF (167).

Since the dominant mechanism of effector cell activation by IL15 *in vivo* is trans-presentation of the IL15Rα/IL15 complex (168), Burkett et al. developed RLI fusion proteins (sushi-IL15Rα and IL15 are connected using a flexible linker). RLIs are functionally more active than IL15 or IL15 plus IL15 plus IL15Rα/IL15 (169), in particular by enhancing cytokine recognition by receptors (170). Development of an IC based on RLI coupled to the C-terminal of the heavy chain of the c.60C3 chimeric antibody against GD2 may increase the half-life due to the small molecular weight of IL15 (169–171). c.60C3-RLI retains the cytokine potential of the fusion protein and the effector functions of the antibody (ADCC and CDC), and its *in vitro* and *in vivo* antitumor therapeutic activity is higher than that of RLI and mAb alone or in combination (172, 173). For example, the combination of dinutuximab, RLI N-803, and NK cells significantly increases antitumor activity (174).

### 3.7 Immunotoxins

In the classical sense, immunotoxins are bifunctional chimeric molecules consisting of an antibody fragment bound to a toxin of plant or bacterial origin (175). Thus, immunotoxins have the antigen-specific properties of an antibody and the activity of a toxin capable of penetrating and destroying a tumor cell by endocytosis (175). In the first studies, full-length mAb 14G2a was combined with plant toxins that inactivated ribosomes, ricin A (176) and gelonin (177). Preclinical studies showed that immunotoxin 14G2a-ricin A can effectively inhibit tumor growth *in vivo* (178, 179). Additionally, immunotoxin 14G2a-gelonin has been shown to be significantly more effective than native gelonin (177). Other immunotoxins, such as those based on scFv mAb 5F11 and diphtheria toxin (180), as well as mAb 14.18 and pseudomonad exotoxin A have also been developed (181). Immunotoxins using the Fv fragment lack the function to mediate ADCC or CDC (180, 181), however, the use of a small antibody fragment promotes better penetration into tumor cells (175). There were no further attempts to develop anti-GD2 immunotoxins, which may be associated with their immunogenicity and major problems in solid tumors. However, the implementation of new approaches aimed at reducing immunogenicity by modifying the structure of toxins or humanizing antibodies, as well as the use of immunomodulatory drugs, may add to the arsenal of strategies (182).

### 3.8 Radiolabeled mAbs and infrared photoimmunotherapy for cancer

Radiolabeled mAbs ^131^I-3F8 were first tested for imaging GD2-positive tumors in mouse models, proving their antigen-specific properties (183). Further clinical application of ^131^I-3F8 demonstrated a significant accumulation of labeled antibodies in high-dose tumors. Scintigraphy with ^131^I-3F8 compared with biopsy, ^131^I metaiodbenzylguanidine (MIBG), and standard diagnostic methods revealed more abnormal sites, including metastases, primarily due to increased sensitivity to neuroblastoma (184). The ^131^I-14G2a antibody was also used for imaging in clinical practice (62), and ^99m^Tc-ch14.18 was more effective in detecting early metastases compared to MIBG (185). On the other hand, mAbs can promote tumor regression, which fits well into the concept of theranostic approach, where labeled antibodies have both diagnostic and therapeutic potential, making radioimmunotherapy (RIT) a feasible approach for the treatment of GD2-positive tumors. The main principles guiding the choice of labeled antibody are high antigen expression and antibody affinity, as well as the biodistribution, pharmacokinetic, and dynamic properties of mAbs (186). This is primarily associated with side effects that particularly affect hematopoiesis and excretory organs. Direct injection of antibodies, e.g. directly into the brain ventricular cavity, is preferred. In particular, this allows anatomical barriers (GEB) to be crossed and the liquor is devoid of leukocytes and proteins that can neutralize mAbs. Clinical trials with intraventricular administration via intrathecal or intraventricular catheter of ^131^I-3F8 (127, 187, 188) showed that the therapy was well tolerated (headache, fever, and vomiting, with no delayed side effects) and can be an adjunct to the main treatment, also in metastatic disease. However, intravenous administration showed no difference in progression-free survival and overall survival between patients receiving 3F8 + GM-CSF + CRA) and ^131^I-3F8 (67). However, this may be explained by stage 4 neuroblastoma complicated by MYCN, which requires further investigation.

Further attempts are made to improve labeled mAbs using different approaches and agents. Thus, multi-step targeting was proposed using the anti-GD2 antibody 5F11 (5F11-scFv-streptavidin) fused to streptavidin and its biotinylated radioactive ligand ^111^In with a DOTA chelating complex that binds mAb and radiolabeled mAb (189). Antigen pre-targeting showed an improved tumor-to-nontumor ratio, but accelerated clearance was observed due to the high immunogenicity of streptavidin. The development of high-affinity scFv to biotinylated DOTA chelator may improve the pre-targeting imaging and therapy strategy (190). Multistep radioimmunotherapy with BiAb, consisting of GD2-targeted hu3F8 and the mouse hapten antibody C825 with high affinity to chelating DOTA in complex with the metals ^177^Lu and ^99^Y, showed a complete antitumor response in a mouse model with minimal toxicity (191). Current imaging techniques rely on positron emission tomography (PET), which has advantages over SPECT in the highly accurate detection of tumors and metastases (192). The antibodies ch14.18/SP2.0 (193), ch14.18/CHO (194), and hu14.18K322 (123, 195) were adapted for PET using the radioactive isotope ^64^Cu in complex with the chelators DOTA, NOTA, SarAr, and their derivatives. The selection of radiopharmaceutical is determined by its safety, stability of the complex, rate of excretion and absorption by tumors and other tissues. For instance, NOTA chelator compared to DOTA binds more stably to ^64^Cu, which can accumulate in various organs and tissues (194, 196). The biodistribution of the 64Cu-SarAr complex after 48 h in the spleen and kidney was shown to be higher than that of other chelator complexes (196), while the safety data are lacking, making clinical application difficult (194). In addition, a decrease in the positive charge of chelators affects biodistribution, in particular, it reduces renal uptake of labeled antibodies (197). There were also no differences in biodistribution and antigen binding between 64Cu-p-NH2-Bn-DOTA in complex with ch14.18 and hu14.18K322 (123, 196). However, their radioimmunologic potential is directly dependent on clinical characteristics and requires further comparative analysis. Subsequent development of labeled anti-GD2 antibodies may focus on the selection of radiolabeled antibodies, chelators, and different antibody platforms (198).

Photoimmunotherapy (NIR-PIT) is a new approach in tumor treatment. It was shown that the GD2 antigen was suitable for this therapy. The essence of NIR-PIT is targeted delivery of anti-GD2 antibody conjugate with photoactivating chemical substance (water-soluble silicon-phthalocyanine derivative near-infrared derivative (IRdye700DX)) followed by exposure to NIR light with a wavelength of 690 nm, which leads to selective cell death (199, 200).

### 3.9 Delivery: mAbs with nanoparticles and drug conjugates

Antigen-specific targeting of anti-GD2 mAbs allows antibodies to be used as transporters of toxic agents and drugs directly into the TME, which may enhance the therapy of solid neoplasms. Conjugated antibodies or their Fab fragments with nanoparticles like radiolabeled mAbs can be used in combination with therapeutic and diagnostic approaches or separately (201). The properties and functions of nanoparticles depend on the material (viruses, lipids, polymers, metals and their oxides, hydrocarbon derivatives, etc.) as well as the antitumor agents loaded in them. Liposomes are spherical phospholipid vesicles capable of penetrating through the tumor vasculature and consolidating at the target site (202). Full-length anti-GD2 mAb and their Fab fragments were conjugated to liposomes loaded with the 13-cis-retinoic acid derivative phenretidine (203), the proto-oncogene suppressing antisense oligonucleotides c-myb (204) and c-myc (205), the chemopreventive agent doxyrubicin (206), siRNAs against vascular endothelial growth factor-A (VEGF-A) (207) and the anaplastic lymphoma caspase (ALK) gene (208, 209), the topoisomerase I inhibitor irinotecan (210) and the sepantronium bromide survivin YM155 (211). Porous silica-based nanoparticles have a homogeneous, inert, and stable structure and a non-toxic safety profile compared to liposomes (212, 213). MAbs ch14.18 bound to porous silica were used to deliver siRNA-34a targeting a wide range of pro-apoptotic genes (213). Iron oxide can be used as a potential binding molecule between the conjugate and mAbs based on catecholamine reactions (214). Non-covalent polymeric carcinostatics (scFv-polymer-carcinostatics) were also shown to be superior in antigen-binding properties and cytotoxic effect compared to covalent ones (215). Carbon nanotube nanoparticles (216) and gold nanorods (217) further enhance mAbs by photothermal degradation when exposed to an infrared laser. Another approach involves the use of compounds of graphene quantum tubes (218) or iron oxide (219) with polyethylene glycol and polyethylenimine, hollow gold particles (220) for tumor diagnosis.

Antibody-drug conjugated (ADC) antibodies consisting of an antibody-linker-drug composition are widely used in cancer immunotherapy (221). Over 80 ADCs are under clinical development, and recent developments are aimed at improving activity, specificity, safety, increasing serum half-life, and decreasing immunogenicity. Compared to immunotoxins, ADCs are less immunogenic, and therefore, less toxic (222). Initial development of anti-GD2 ADC using 14G2a and a synthetic analog of calicheamicin showed significant suppression of liver metastases in a mouse model (223). It is noteworthy that until recently, there were no conducted studies, although ADC-based therapies showed good antitumor responses. However, after 20 years, an ADC based on ch14.18 and monomethylauristatin E (MMAE) and F (MMAF) was developed that showed potent antitumor activity with the antibodies retaining stability, antigen-binding properties, and *in vivo* biodistribution profile (224), making this a promising area for further study. It has been shown that higher antigen density leads to a stronger internalization of mAbs (225). Therefore, MMAF-conjugated mAbs will be more effective in killing tumor cells with high GD2 density, as MMAE penetrates tumor cells better than MMAF (224). The development of antibody fragments, so-called minibodies, based on ch14.18 (two scFv linked by a linker to the CH3 domain of IgG1) conjugated to MMAE and MMAF (FDC), is also reported (226). The results show the therapeutic potential of FDC compared to ADC, including improved pharmacokinetic characteristics, reduced side effects associated with the absence of Fc-fragments, and pronounced cytotoxic properties.

Internalization of anti-GD2 antibodies can provide a means to deliver drugs or toxins directly into the tumor cell. However, it can also be a mechanism for tumors to evade immunotherapy with naked mAbs. Conjugating mAbs with endocytosis inhibitors, such as EIPA (5-(N-ethyl-N-isopropyl) amiloride), chlorpromazine, MBCD (methyl beta-cyclodextrin), and cytochalasin-D, has shown potential to inhibit antibody internalization (225). In addition, MBCD-conjugated mAb can enchance ADCC that may improve the efficacy antitumor therapy.

### 3.10 GD2 aptamers

In addition to mAb, “chemical antibody” aptamers were developed, which are single-stranded DNA or RNA molecules selected by an iterative selection process called systematic ligand evolution by exponential enrichment (SELEX) (227). High affinity aptamers recognize the GD2 antigen, so they can be conjugated to other molecules and toxins for drug delivery or imaging (228, 229). The main advantages of aptamers over mAbs include small molecular size and high permeability through blood vessels and GEB, high affinity, non-immunogenicity, safety, and low cost. At the same time, the structure of the molecules can be easily synthesized and modified for various therapeutic purposes due to geometric conformational flexibility and synthetic dynamics (229, 230). To date, two GD2 aptamers with doxirubicin incorporated into the structure were developed, one containing a pH-sensitive motif to reduce side effects and the ability to be activated in an anaerobic environment by TME (DB67) (229), and the other – by MYCN-siRNA (DB99) (230).

### 3.11 Bispecific antibodies

Bispecific antibodies (BiAbs), compared to classical antigen-specific antibodies, are able to recognize TAAs and additionally attract cytotoxic cells by targeting costimulatory molecules or receptors (231). Bispecific T-cell activators (BiTE), compared to BiAb, typically consist of two scFv as a polypeptide chain, with the light and heavy chains connected to a flexible linker (232). Various BiAb constructs targeting GD2 and CD3 were tested in preclinical models. In particular, BiAb were obtained by fusing IgG anti-GD2 antibody with scFv anti-CD3 antibody (233), chemical heteroconjugation of mAbs anti-GD2 and anti-CD3 (178, 234), scFv anti-GD2 with scFv anti-CD3 (BiTE) (22), which demonstrate binding of GD2-positive tumors and activated T cells in an MHC-independent manner, exhibiting cytotoxic properties through the perforin/granzyme axis. The hu3F8-based BiAb was shown to induce rapid T cell infiltration and expansion, mediating potent T cell-dependent cytotoxicity (TDCC) (235). Adoptive transfer of *ex vivo* proliferated T cells armed with GD2-BiAb leads to rapid tumor infiltration and induces a potent antitumor response (GD2-EAT) with significantly lower production of cytokines that induce CRS (236). At the same time, over-activation of T cells by BiAb may be resolved by aglycosylation of IgG-scFv (233). Combination treatment with the checkpoint inhibitors pembrolizumab (PD-1) or atezolizumab (PD-L1) enhanced armed BiAbs T-cell function and tumor control when administered sequentially and continuously (237). BiAb-directed T cells demonstrate superior cytotoxic properties and are less depleted than GD2.CAR-T cells (238). The present studies are aimed at optimizing the structure of BiAbs, taking into account the size of the constructs and affinity to tumor antigens, which affects biodistribution and cytotoxicity *in vitro* and *in vivo*. Thus, it was shown that for anti-GD2 BiAbs, the optimal option was to place the antigen and T-cell binding domains in a cis-configuration with a two-wall IgG-[L]-scFv platform and the use of two cis-modules additionally increased cytotoxicity (239). The rapid half-life of BiAbs requires continuous administration, which can be solved by increasing the molecular weight of the antibodies, in particular, by using tetravalent antibodies with two binding sites (240) or in complex with metals (191).

Trifunctionalized BiAbs (TrAbs) consist of heterodimeric isotopes of murine IgG2a and rat IgG2b. Their function is enhanced by the presence of an Fc region, which provides high affinity binding via FcγR to antigen-presenting cells (APCs) in addition to T cells; in particular, dendritic cells, monocytes, macrophages (241, 242), and lower affinity to NK cells (243). In mouse models, TrAbs SUREK-based vaccines were shown to promote T-cell recognition of TAAs (244), as well as the development of humoral response, in addition to a given GD2 antigen (242). In addition, treatment with anti-GD2 TrAbs SUREK was superior to dinutuximab beta against neuroblastoma (245), and when combined with an antitumor vaccine and immune checkpoint inhibitors, it stimulated the endogenous response and enhanced the antitumor effect (243). Due to low binding to the GD2 antigen (242), TrAb can be further utilized as an additional boost to the main therapy.

## 4 Anti-GD2 and idiotopic vaccine

The basic idea behind antitumor vaccines is to create a specific immune response in response to TAA administration. By nature, GD2 is a carbohydrate antigen. Thus, to enhance immunogenicity, strong protein-framed adjuvants such as keyhole limpet hemocyanin (KLH) (246) or non-toxic diphtheria toxin CRM197 (247) followed by subcutaneous injection of Quillaja saponaria (QS) (248) or monophosphoryl lipid A (249) are needed to enhance the cellular response (250). Active immunization of patients with GD2-KLH/MPL-A did not induce antibody formation against GD2 (249), and despite the serologic response from GD2-KLH/OPT-821 (equivalent to QS-21), there was no significant difference in progression-free survival between the control and subject groups (248). However, the right approach to vaccine development could potentially improve therapy. Subsequent trials of a bivalent GD2/GD3-KLH/OPT-821 vaccine combined with oral administration of β-glucan (a C-type lectin receptor activator) showed encouraging results with no serious toxicity (251); and subsequent immunization of an expanded cohort demonstrated a strong humoral response, with a high anti-GD2-IgG1 titer associated with better survival (252).

There were also attempts to develop idiotypic vaccines, also knows as anti-Id vaccines. The fundamental concept behind these vaccines is to prolong a humoral or cellular response by using anti-Id vaccines against already developed anti-idiotypic antibodies after previous therapy with anti-GD2 mAbs (253). Since TAAs are autoantigens, especially carbohydrate antigens, there is a tolerance of immune response to them, so the use of anti-Id vaccines would be able to overcome this barrier (254). Anti-Id mAbs murine 1A7 against ch14.18 (255) and rat A1G4 against 3F8 were developed (256). Clinical use of 1A7 showed no toxic effects, but objective responses were minimal (255). However, a ganglidiomab antibody against anti-GD2 antibody family 14.18 was later developed, which induced a humoral response in murine models (257) and among patients after therapy with anti-GD2 mAbs, demonstrating good tolerability without significant side effects (258). The development of anti-Id antibodies mimicking human and mouse GD2 ganglidiximab, which is capable of mediating ADCC and CDC, and which may be useful for tailoring humoral responses to paratopic regions mimicking GD2, was also reported (259).

## 5 Cell therapy

Cell-based immunotherapy involves the adoptive transfer of GD2-targeted genetically modified, virally vector-mediated (retroviral or lentiviral), or non-viral approaches (sleeping beauty transposition), or *ex vivo* stimulated NK-, NKT-, and T-cells in combination with anti-GD2 mAbs and other drugs for chemotherapy. Chimeric antigen receptor (CAR) cells are suitable for GD2-targeted therapy because they have unique properties to recognize targets of different classes, including glycolipids and carbohydrates, which have lower mutation rates (260). CAR recognizes the target in an MHC-independent manner using a single-chain variable fragment (scFv) derived from mAb. Since the construct includes costimulatory domains (CD27, CD28, 4-1BB, ICOS, OX40, and etc.), cells activated after encountering the CAR antigen do not need additional stimulation. This chapter presents different approaches and strategies to improve CAR therapy (**Table 1**), in particular through combination therapy and gene modification of different effector immune cell populations (**Table 3**).

**Table 3:** Comparison of αβ T, γδ T, NK, NKT cells and macrophages with CAR. Current clinical trials of anti-GD2 CAR therapy.

	CAR-αβ T cells	CAR-γδ T cells	CAR-NK cells	CAR-NKT cells	CAR-M				
Source Expansion	PBMC Anti-CD3/CD28 and IL-2/-7/-15	PBMC Anti-CD3/CD28 and IL-2/-7/-15 plus ZOL, ConA or PTA	PBMC, UCB, BM, hESC, HSPC, iPSC or NK-92 cell line IL-2/-12/-21 and/or K562 feeder cells with membrane-bound IL-21 and 4-1BBL	PBMC, UCB, BM, HSPC and iPSC Magnetic sorting plus anti-CD3/CD28, IL-2/-7/-15/-21, α-GalCer-pulsed APC or feeder cells	PBMC, UCB, BM, HSPC and iPSC M-/GM-CSF, IL-1β, IFN-γ, and lipopolysaccharide for M1 polarization			
CAR structure Receptors	ζ-chain and CD27, CD28, 4-1BB, ICOS and OX40 domains αβ TCR	ζ-chain and domains of T and NK cells γδ TCR, FcRs, NKRs	ζ-chain and domains of NK (2B4, DNAM1, DAP10, DAP12) and T cells (CD28, 4-1BB) NKRs	ζ-chain and domains of T cells Semi-variant αβ TCR, NKRs	ζ-chain (homology with FcϵR1-γ), TLR (2, 4, 6), MerTK, Megf10 or domains of T cells TLRs; FcRs			
Features	MHC-independent TAA recognition Heterogeneous population of T cells Simpler to obtain and expand Memory phenotypes Clinical use is widespread	MHC-independent recognition of a wide range of TAA (proteins, lipids, etc.) Properties of T cells, NK cells and APCs Cross-presentation of antigen to αβ T cells Interaction with B cells and switch Ig classes Strong cytotoxic activity Reduced CRS and GvHD	Do not need to pre-sensitize Strong cytotoxic activity Reduced CRS and GvHD, mild adverse effects	Recognition of MHC I-like CD1d molecules Stimulation of immune system cells and suppression of TAMs and MDSCss Properties of NK cells and APC Cross-presentation of antigen Reduced GvHD	High infiltration of TME Stimulation and recruitment of immune system cells Professional APC ECM remodeling Reduced GvHD			
Activation and cytotoxic mechanisms	Activation by antigenic stimulation of CARs and built-in costimulatory signals Perforin/granzyme axis, Fas/FasL apoptosis, proinflammatory cytokine release	Activation and cytotoxic mechanisms of CAR-T cells NK cell toxicity receptors NKG2D (NKp30, NKp44, and NKp46) ADCC	CAR-dependent/independent cytotoxicity regulated by stimulatory and inhibitory signals Perforin/granzyme axis, Fas/FasL or TRAIL apoptosis, proinflammatory cytokine release ADCC	CAR-dependent/independent cytotoxicity Cytotoxic mechanisms of CAR T cells and NK cells toxicity receptors	CAR-dependent/independent cytotoxicity Proinflammatory cytokine release and toxic molecules (ROS, iNOS, NO) Phagocytosis and ADCP			
Limitations	Cytotoxicity limited by TAA expression Suicide gene required Adverse effects: CRS, immune effector cell-associated neurotoxicity syndrome (ICANS), non-tumor toxicity, GVHD	1-5% of circulating cells Low clonal expansion, persistence/survival, and longevity	10-15% of circulating cells Limited proliferation	About 1% of circulating cells Difficult to expand and obtain Low TME infiltration	About 6% of circulating cells Limited proliferation and efficiency of transduction, highly resistant to genetic modifications Risk of polarization into M2 due to TME effects Low clinical use			
GD2-targeting cell therapy: current clinical trials									
CAR-T cells	(active, not recruiting) NCT01953900 NCT03635632 NCT01822652 NCT00085930 (recruiting) NCT05437315 NCT03373097 NCT04099797 NCT05438368 NCT05437328 NCT05298995 NCT05544526 NCT05620342 NCT04637503 NCT03721068 NCT04196413 NCT04430595	iC9-GD2-CAR-VZV-CTLs GD2-C7R-T cells iC9-GD2-CD29-OX40 T cells iC9-GD2-CAR-EBV-CTLs bi-4SCAR-GD2/PSMA iC9-GD2-CART01 GD2.C7R-CAR bi-4SCAR-GD2/CD70 bi-4SCAR-GD2/CD56 iC9-GD2-CAR T-cells GD2CAR T-cells iC9-GD2.CAR.IL-15 T cells 4SCAR-T cell iC9-GD2-CAR-IL-15 T cells iC9-GD2-BBz-CAR T cells 4SCAR-T cells targeting Her2, GD2, and CD44v6	CAR-T cells plus VZV vaccine CAR-T cells with chemotherapy CAR-T cells with chemotherapy plus pembrolizumab CAR-T cells Bi-specific CAR-T cells CAR-T cells CAR-T cells infusion intravenously and directly into the brain Bi-specific CAR-T cells Bi-specific CAR-T cells CAR-T cells CAR-T cells intraventricular catheter infusion with chemotherapy CAR-T cells Combinational GD2/PSMA/СВ276 CAR-T therapy CAR-T cells with chemotherapy CAR-T cells with chemotherapy Multi-CAR-T cells	Sarcoma, Neuroblastoma Neuroblastoma, GD2^+^ tumors Neuroblastoma Neuroblastoma Solid tumors Neuroblastoma, Solid tumors Brain tumors GD2 and/or CD70^+^ tumors GD2 and/or CD56^+^ tumors CNS tumors DMG Lung cancer Neuroblastoma Neuroblastoma, osteosarcoma DIPG, DMG Breast cancer	Phase I Phase I Phase I Phase I Phase I/II Phase I/II Phase I Phase I/II Phase I/II Phase I Phase I Phase I Phase I/II Phase I Phase I Phase I/II			
CAR-NKT cells	NCT03294954 (recruiting)	GD2-CD28-CAR-IL-15 NKT cells	CAR-NKT cells with chemotherapy	Neuroblastoma	Phase I			

Peripheral blood mononuclear cell (PMBC), umbilical cord blood (UCB), bone marrow (BM), human embryonic stem cells (hESC), hematopoietic stem/progenitor cells (HSPC), induced pluripotent stem cells (iPSC), antigen-presenting cells (APCs), MDSCs (myeloid-derived suppressor cells), tumor-associated macrophages (TAMs), Epstein-Barr virus-specific cytotoxic T lymphocytes (EBV-CTLs), Varicella-Zoster virus-specific cytotoxic T lymphocytes (VZV-CTLs), diffuse intrinsic pontine glioma (DIPG), diffuse midline glioma (DMG), central nervous system (CNS), tumor-associated antigen (TAA), prostate-specific membrane antigen (PSMA), cytokine release syndrome (CRS), graft versus host reaction (GvHD), immune effector cell-associated neurotoxicity syndrome (ICANS), extracellular matrix (ECM), tumor microenvironment (TME), major histocompatibility complex (MHC), antibody-dependent cell-mediated cytotoxicity/antibody-dependent cellular phagocytosis (ADCC/ADCP), complement-dependent cytotoxicity (CDC), nitric oxide synthase (iNOS), reactive oxygen species (ROS), nitric oxide (NO), constitutively activated IL-7 receptor (C7R), inducible caspase 9 (iC9), concanavalin A (ConA), tetrakis-pivaloyloxymethyl-2-(thiazole-2-ylamino)ethylidene-1,1-bisphosphonate (PTA), zoledronate (ZOL).

### 5.1 CAR-T cells

The cytotoxic potential of CAR-T cells is widely used in clinical practice, and, unlike TCR, it is realized by the formation of a non-classical immune synapse that has an advantage in kinetics and enhanced signal transduction with comparable amounts of lytic molecule release (261). The main antitumor effects of CAR-T cells are realized through the major cytotoxic axis of perforins and granzymes (targeting antigen-positive fraction), as well as through the Fas/FasL axis (targeting antigen-negative fraction) and the release of cytokines (stromal cell sensitization) such as IL2, IL15, IFNγ, TNFα, etc. (262) (**Figure 1**). The expression profile of surface markers affects clinical responses. At the same time, CAR-T cells with a memory-like phenotype (CD62L, CCR7, CD45RA and CD45RO) provide high antitumor efficacy, whereas acquisition of a depleted cell phenotype (PD-1, LAG-3 and TIM-3) is associated with limited efficacy (263). It was also found that targeting GD2 with CAR-T compared to mAb had several advantages: 1) CAR polyvalency on the surface of T cells may have a higher overall avidity than a soluble antibody in divalent form, thereby increasing the probability of binding to tumor cells with lower GD2 expression; 2) additional cytotoxic mechanisms of T cells allow for more efficient destruction of tumor cells; 3) the duration of T cell persistence in the circulation may provide relapse control (264); CAR-T cells have the ability to cross the blood-brain barrier (265) compared to mAbs (58). However, despite their clear therapeutic potential, CAR-T may be limited by their rapid loss of functional properties and development of a depletion stage, as well as by their low *in vivo* proliferation and ability to infiltrate the tumor, which may be associated with immunosuppressive TME and its extracellular matrix, and lack of co-stimulatory stimulus when interacting with tumor cells. AICD (activation-induced cell death), which may be mediated by antigen re-stimulation (266) or Fas-FasL interaction, is suggested to be another limitation of CAR-T cell function (267). Nevertheless, excessive functional CAR-T activity is often associated with the manifestation of side effects including neurotoxicity, cytokine release syndrome (CRS), and GvHD (268). In addition, CAR-T cells are limited in large-scale individualized preparation. Given this series of challenges, strategies to improve CAR-T therapy focus on various modifications of the CAR structure as well as the route of delivery and targeted delivery.

### 5.2 Design of GD2.CAR cells

Functional properties and stability in the body depend on CAR design, including scFv, spacer and costimulatory domains, as well as additional components that enhance cell performance. First-generation GD2 CAR studies have demonstrated the importance of CD28 costimulatory signaling in specific antigen recognition for T cell survival and expansion, as well as for enhancing the immune response, including through IL2 secretion (269). However, the presence of the antigen-binding domain and CD3 ξ-chain alone does not provide sufficient stimulus to ensure the functional properties of CAR-T cells (270). An alternative mode of activation was based on the physiological stimulation of native TCR through interaction with APCs, which was achieved by transduction of virus-specific T cells (271, 272). The persistence of EBV-specific GD2.CAR-T cells was shown to be longer compared to autologous activated GD2.CAR-T cells (273). However, in the long term, despite the presence of antitumor efficacy with simultaneous CAR and TCR stimulation, virus-specific CAR-T cells was less retained in the bloodstream (264).

The costimulatory domains CD28 and 4-1BB (CD137) are the most commonly incorporated into CAR constructs, with T cell functions depending on domain selection. The CD28 domain was shown to enhance proliferation and IL2 release (274), and is a more potent driver of antitumor response compared to 4-1BB (275). In addition, the CD28 domain also enhances cytokine production and cytotoxicity for CARs with low avidity by lowering the threshold of antigen affinity (276). In contrast, the inclusion of the 4-1BB domain is associated with increased persistence, proliferation (277), and potent therapeutic activity *in vivo* (278). Antigen-independent signaling can induce earlier depletion of CAR T cells, with the CD28 domain increasing key aspects of depletion, while in contrast, the 4-1BB domain can improve antitumor effects by producing higher levels of cytokines, reduced expression of depletion markers, and increased resistance *in vivo* regardless of antigen-dependent or independent effects (277). However, it was shown that 4-1BB-based tonic CAR signaling could induce T cell apoptosis through continuous TRAF-dependent activation of the NFκB pathway and Fas-dependent cell death (279). Transcriptional analysis showed that GD2.28z.CAR T cells exhibited higher expression of genes encoding inhibitory receptors such as LAG3, HAVCR2 (TIM-3), CTLA4, BTLA, and CD244 (2B4), and the depletion-related transcription factors *TBX21 (T-bet), EOMES, PRDM1 (Blimp-1)*, and *IKZF2* (Helios) compared to GD2. BBz.CAR T cells that express memory-related transcription factors such as *KLF6, JUN*, and *JUNB* (277).

Tandem use of costimulatory domains can provide improved signal transduction as well as compensate for the deficiencies of a single domain. Thus, it was shown that the use of the CD28 domain alone could not sustain prolonged growth, activity, and survival of T cells (269). The OX40 domain (CD134), which is expressed after T cell activation following antigen and CD28 stimulation, is required for ongoing proliferation and cytokine production (280, 281). It is also noted that the replacement of OX40 with the 4-1BB domain or ligand can reduce or prevent AICD and/or PD-1-mediated suppression (266). It was shown that GD2.CD28.OX40z T cells resulted in maximal NF-κB activation associated with increased and prolonged proliferation and enhanced cytokine release compared with the inclusion of CD28 or OX40 alone (282). However, in another study, GD2.CD28.OX40z secreted less INFγ and IL2 after 30+ days, and their phenotype correlated with exhaustion status compared to GD2.4-1BB.CD28z (283). Phosphoproteomic analysis also showed that GD2.CD28.OX40z had the highest number of phosphorylation sites, suggesting that the cells were overstimulated. The ICOS domain (a member of the CD28 family) can also be added to the CAR construct to enhance the antitumor activity of CD8+ T cells by differentiating CD4+ T cells into the Th1/Th17 phenotype (284). In addition, the ICOS domain has a better effect on CAR-T survival compared to CD28 and also complements the function of the 4-1BB domain, including reducing tonic signaling (284), which likely contributed to the better persistence and antitumor activity of GD2 CAR-T cells *in vivo* (285). At the same time, combined stimulation of CD28 and 4-1BB may contribute to cytokine storm due to forced stimulation of CD28 (286). However, for third-generation GD2 CAR-T cells, the combination of CD28 and 4-1BB domains is the most optimal. 4-1BB signaling promotes the restoration of CD28-induced depletion and the most homogeneous distribution of CARs on the cell surface, with GD2.4-1BB.CD28z exhibiting effective antitumor activity *in vivo* (283). For virus-specific CAR-T cells, it was shown that the most optimal domain is CD28, as GD2.CD28z better supports the TCR function (287).

Chimeric TCR signaling is more efficient when mediated by the ξ-chain compared to the γ-chain FcϵRI (270). The choice of the variable fragment is dictated by the conditions of optimal affinity and high specificity of antigen recognition; therefore, ch14.18 is often used as scFv. However, scFv derived from 14g2a causes rapid depletion of GD2.CAR-T cells due to tonic signaling during *ex vivo* expansion (277). A humanized antibody can be used as a substitute for ch14.18. Thus, it was shown that scFv derived from the humanized antibody KM8138 did not cause anti-idiotypic rejection of CAR-T cells with preservation of their functional activity (288). In addition, scFv derived from hu3F8 allowed CAR-T cells to better target the tumor and promoted increased cytolytic activity compared to scFv based on mAbs CE7 and 14g2a (289, 290). It was also reported that the inclusion of a mutation in the spacer Fc domain avoided off-target toxicity by reducing high-affinity FcγR binding (280), as it can prevent binding to γ receptors of immune cells (291). In a preclinical model, this strategy provokes high neurotoxicity despite high *in vivo* efficacy (265, 292), making it not feasible.

Altering tonic signaling by reducing positively charged CAR sites through mutations or increasing ionic strength (increasing pH in the culture medium due to carnosine during *ex vivo* propagation) improves efficiency and reduces the fatigability of GD2.CAR-T cells (293). Application of PI3K (294) or Akt-pathway (295) inhibitors can block tonic CAR signaling at the initial stage of preparation, while additionally reducing terminal T cell differentiation. A strategy to reduce GD2.CAR-T depletion can be aimed at temporarily halting CAR signaling by turning on the C-terminal destabilizing domain of FK506 binding protein 12 (FKBP) using a drug-regulated system or the multikinase inhibitor dasatinib (296). Tonic signal transduction can be reduced by altering the TRAC locus, and cells have a delayed ability to differentiate *in vitro* and *in vivo* (297).

### 5.3 CAR-T cells and TME

Overcoming immunosuppressive and heterogeneous TME of solid tumors is one of the leading tasks to achieve the efficiency of GD2.CAR-T cells. Several approaches were used to realize this goal, including those aimed at increasing cell migration and enhancing cell cytotoxic properties. TRUCK CARs (“T cells redirected for antigen-unrestricted cytokine-initiated killing”) have the ability to produce transgenic pro-inflammatory cytokines IL7, IL12, IL15, IL18, IL23, and their combinations by CAR signaling induced by NFAT (nuclear factor of activated cells) (298, 299). TRUCKs GD2.CAR T cells secreting IL18 (300) or IL12/18 (301), were shown to have enhanced effector properties and also promote monocyte recruitment to the tumor. Co-expression of transgenic IL15 significantly increased GD2.CAR-T engraftment and also promoted additional sustained tumor control (292). In addition, IL15 also promotes differentiation into memory and stem cell-like phenotypes, with GD2.CAR-T exhibiting reduced PD-1 expression and increased survival in both peripheral blood and tissues (302). GD2.CAR T cells co-expressing chemokines IL7 and CCR2b were also developed, which had chemotaxis ability, improved proliferation and survival *in vivo* in addition to strong antitumor activity (303).

The combined use of CAR-T cells and oncolytic viruses (OVs) also aims to immunomodulate TME. OVs can be delivered by CAR-T cells to tumors systemically and provide direct lysis, or generate *in vivo* expansion of T cells with native TCR specificity to viral or virus-encoded antigens and enhance antitumor activity by inducing phenotypic changes in T cells with dual specificity (304). In addition, OV armed with various chemokines and cytokines can be used for CAR-T therapy. It was shown that the use of GD2.CAR-T cells and OVs armed with the chemokine RANTES and IL15 directly accelerated caspase pathways in tumor-exposed T cells, with RANTES and IL15 promoting CAR-T recruitment to the tumor and ensuring their local survival (305). In order to overcome the lack of immunogenicity of solid tumors, modification of GD2.CAR-T cells (NCT01953900) specific to varicella-zoster virus (VZV CAR-T) can restore cell function by preserving sensitivity to stimulation via TCR either by VZV vaccine or after co-culture of VZV CAR-T and APC treated with VZV peptides (306).

Tregs, MDSCs, tumor-associated macrophages (TAMs) M2, immune checkpoint molecules (PD-1 and CTLA-4), and growth factors and anti-inflammatory cytokines and chemokines are the main components of TME (307), targeted by CAR-T therapy in combination with drugs. It was shown that when tumors were re-expressed with GD2.CAR-T cells, there was increased expression of PD-1 and PD-L1 inhibitory molecules, requiring therapy with immune checkpoint inhibitors (290). Preclinical trials of GD2.CAR-T cells with nivolumab (a PD-1 inhibitor) (308) and bevacizumab (a vascular endothelial growth factor VEGF inhibitor) (309) demonstrated the efficacy of the combinations, which could be used in clinical practice. However, the combination of GD2.CAR-T cells with pembrolizumab (PD-1 inhibitor) did not show to have an expressed effect in patients with neuroblastoma, which may be related to the timing and duration of PD-1 inhibition and tumor type (310). In particular, pembrolizumab and nivolumab were shown to be particularly effective against melanoma and lung cancer (311). In addition, the positive antitumor effect of PD-1 blockade is directed to the inhibition of AICD (266). Inhibitory MDSCs are an obstacle in the antitumor response and may worsen the prognosis for patients with cancer (310). In preclinical models, GD2.CAR-T cells did not exert antitumor effects, which was likely to be associated with the inhibition of human cells by murine MDSCs (312). Patients’ initial PBMCs may also be a barrier to CAR-T cell expansion at the initial stage (313), including because of MDSCs that suppress the expression of genes involved in cell activation, signal transduction, inflammation, and secretion of cytokines and chemokines (314). Combination with trans-retinoic acid (ATRA) may improve the antitumor activity of GD2.CAR-T cells by reducing the suppressor effect of MDSCs (314). IGF1R/IR inhibitors (linsitinib) show synergism with GD2.CAR-T cells (315), while its use can inhibit Treg (316) and M2 macrophage differentiation (317). Supplying GD2.CAR-T with additional GITRL expression may also enhance T cell efficiency in TME (318). The development of CAR-T cells that produce antibodies to PD-L1 was shown to reduce tumor growth in a mouse model (319).

The FDA-approved BRAF (dabrafenib, vemurafenib) and MEK (trametinib, cobimetinib) inhibitors aim to stop MAPK signaling leading to unregulated cell growth and differentiation, and their benefits for the treatment of solid tumors were shown in clinical practice (320). Combining CAR-T cells with BRAF/MEK inhibitors has the potential to be a new effective treatment option, but there is a question about the effect of inhibitors on T cell function. The dabrafenib/trametinib combination was shown to have no effect on the cytotoxic functions of CAR-T cells compared to vemurafenib (321) or the vemurafenib/cobimetinib combination (322). In addition, the combination of GD2.CAR-T cells and trametinib improves *in vivo* and *in vitro* antitumor efficacy compared to cell monotherapy, in particular by suppressing T cell depletion as well as reducing PD-L1 expression on neuroblastoma cells (323). The PD-1/PD-L1 axis can be blocked by doxorubicin, which also enhances the cytotoxic effect of GD2.CAR-T cells (324). Conditioning with cyclophosphamide/fludarabine (Cy/Flu) (lymphodepletion) also shows antitumor efficacy, including by an increase in CAR-T cells proliferation (310, 325).

The problem of the heterogeneous structure of solid tumors can be solved by targeting multiple target antigens. In particular, sequential administration of CD171- and GD2-specific CAR-T cells enhanced antitumor response and helped to prevent antigen loss in preclinical trials (289). Targeting GD2 and HER2 can be combined in a single bispecific CAR (TanCAR) consisting of two separate linked scFv domains for TAA recognition, which also compensates for antigen escape (326). The addition of tazemetostat to the treatment regimen may increase the expression of GD2 antigen, and therefore, increase susceptibility to targeting (327). In the future, BiTE-secreting CAR-T cells (328, 329) may be developed for GD2.CAR-T therapy to effectively kill heterogeneous tumors.

### 5.4 Production of CAR-T cells, safety, and delivery

Another promising direction for CAR-T technology is to produce CARs without viral transduction, which may have advantages in production, facilitating monitoring of vector replication ability, and eliminating accidental integration of viral elements into the human genome (297). Viral transduction of T lymphocytes also results in the proliferation of not only CAR-T cells but also CAR-NK cells (330). GD2.CAR-T cells were successfully generated using the piggyBac (323) and CRISPR/Cas9 systems (297). CAR-T cells derived from CRISPR/Cas9 had a less depleted phenotype compared to retrovirus-transduced cells (297). All-in-one lentiviral constructs with a single vector utilize a single vector integration event, which also reduces the potential risk of a potential mutagenesis side effect (301). In addition, lentiviral vectors can transduce cells regardless of their division status, whereas retroviral vectors can do it only during mitosis (331). The use of the CliniMACS Prodigy device allows for the large-scale production of finished GD2.CAR-T cells (300, 332), which greatly simplifies the production of the final product.

Prolonged culturing during the production stage can cause earlier depletion of CAR-T cells. Initial stimulation leads to potent production of INFγ and TNF, and loss of IL2 secretion is identified as the first stage leading to depletion (266). The use of the GDAIN protocol improves the survival of GD2.CAR-T cells and promotes differentiation of central memory or naive/stem-like T cells (effector memory phenotype is associated with a poor antitumor response). GD2.CAR-T production by apheresis and elutriation (washed lymphocytes) is better than by magnetic sorting of anti-CD3/CD28 or adhesion to anti-CD3/CD28 plastic, which significantly affects CAR-T quantity and quality (333). The combination of CD3 and CD28 with IL7 and IL15 gives the best balance of CAR-T expansion and potent effector cells while maintaining the stem/memory phenotype (stem/memory subset – CD45RA, CCR7, and CD95) (334).

Integration of an inducible “safety switch” (iCasp9) into the CAR construct allows the removal of mis-activated cells to avoid excessive off-tumor toxicity as well as CRS and MAS (335). GD2.CAR-T cells with iCasp9 were tested in various clinical trials (302, 324, 336, 337), noting that the treatment was safe with minimal side effects. The UniCAR platform ensures safety by adding a specific on/off module, thus avoiding off-target toxicity in the periphery (338). Local administration of CAR-T cells is not only effective but also safe compared to systemic administration (339–341). The delivery of GD2.CAR-T cells encapsulated in chitosan-PEG *in situ* injectable hydrogel is an excellent solution for the treatment of retinoblastoma to reduce inflammation and prevent retinal detachment (341).

### 5.5 GD2-targeting CAR and adoptive therapy: NK, NKT, γδ T cells, and macrophages

NK cells are part of the innate immune system responsible for protecting the body from malignancy. Unlike T cells, which are MHC-restricted and require sensitization and the presence of a tumor target, NK cells are able to rapidly activate and destroy tumor cells through direct cytotoxicity, formation of proinflammatory cytokines and chemokines, as well as by manifesting ADCC through the membrane receptor FcγRIII (CD16) or the apoptosis axis through TRAIL or Fas/FasL (43, 44). NK cell activity is regulated by both activating signals (DNAM-1, NKG2D, CD226, NKp30, NKp44, NKp46, etc.) and inhibitory signals (KIR, CD94/NKG2A, TIGIT, etc.) through the interaction of cell membrane receptors with ligands on target cells (342, 343). It was shown that the effect mAbs exert on NK cells was not limited to ADCC, with FcγRIII-mediated signaling being able to block KIR inhibition (344). NK cell therapy with NK cells achieved considerable success in tumor regression and disease stabilization, and one of its major advantages is the absence of GvHD, making it attractive for allogeneic transfer (345). Preclinical studies showed that the antitumor activity and functional properties of NK cells could be further enhanced when combined with ch14.18 (93, 346, 347), hu14.18-IL2 (348), and galunisertib (TGFβR1 inhibitor) (93), IL2 and IL15 (349), IL21 (346, 347). The addition of IL21 enhances ADCC, activating receptor expression and granzyme release, while galunisertib has a remodeling effect on TME. In addition, cytokine-induced killer cells using IL2 and IL7 in combination with anti-GD2 can significantly increase the rate of cell death compared to the treatment with each of them separately (350). Clinical trials with adoptive transfer of haploidentical NK cells in combination with hu14.18K322A, GM-CSF and IL2 (126, 351), m3F8 (70) show promising results. In addition, the toxicity profile associated with mAbs was not altered by the administration of NK cells indicating their safety.

A large share of studies in GD2.CAR therapy focused on T cells, but its efficacy was hampered by TME, side effects, and the associated cost of treatment. From this point of view, it was hypothesized that NK cells had several advantages and might become better CAR drivers than T cells (352). NK cells are safer, do not cause GvHD and other side effects, produce mainly INFγ and GM-CSF (unlike T cells that induce CRS by TNFα, IL1, and IL6), can be activated by a variety of receptors, and are able to mediate ADCC. The NK-92 cell line is used to develop CARs, including GD2.CAR-NK cells. NK-92 is believed to be an ideal CAR host because it has natural antitumor properties and is easy to scale and modify (353). However, NK-92 cells cannot mediate ADCC because they lack CD16, carry an abnormal genome, and are irradiated before use, which may reduce their potential. Therefore, other sources of NK cells, (e.g. pluripotent stem cells) are required to test the hypothesis for GD2. Nevertheless, preclinical studies showed that GD2.CAR-NK cells could effectively kill tumor cells *in vitro* (354, 355) and *in vivo* (356, 357), as well as enhanced INFγ production (357). In addition, armored GD2.CAR-NK cells expressing IL12 show tendencies to recruit monocytes (355).

T cells with natural killer cell properties (NKT cells) and γδ T cells combine the innate and adaptive properties of the immune response and represent a subset of T cells that express different receptors, including those characteristic of NK cells (358). γδ T cells are characterized by expression of heterodimeric TCRγδ, whereas NKT cells express semi-invariant TCRαβ. The direct mechanism of NKT-cell and γδ T-cell cytotoxicity includes perforin/granzyme B-mediated cytolysis, TNF and TRAIL production, and Fas/FasL-dependent apoptosis (359, 360). NKT cells are characterized by reactivity to glycolipids of their own and microbial origin via the MHC I-like molecule CD1d and the α-GalCer glycolipid antigen presented by it (361). In addition, NKT cells exert potent antitumor potential by stimulating NK- and dendritic cells and priming αβ T cells (362), rapid and efficient migration to TME (363) and suppressing TAM and MDSC immunosuppression (364). In turn, γδ T cells can act as APCs for T cells at the tumor site (365), kill the tumor via ADCC and FcγRIII (CD16), and interact with B cells and switch Ig classes (46).

Their antitumor potential and ability to recognize a wide range of antigens, exert direct and indirect cytotoxicity, and influence immunosuppressive TME make NKT- and γδ T cells potential candidates for GD2-specific CAR therapy. Unlike CAR-T cells, their activation does not depend on CAR signaling because they can recognize antigens in an MHC-independent manner, and therefore, do not induce GvHD responses. Despite few studies, GD2.CAR-γδ T cells showed to be capable of antigen cross-presentation, leading to clonal expansion of αβ T cells, with cytotoxicity equivalent to GD2.CAR-αβ T cells (366). In addition, GD2.CAR-γδ T cells can target tumors with low antigen density compared to CAR-αβ T cells (367). Replacing the ξ-chain with DAP10 (chimeric costimulatory receptors) downregulates tonic signaling with preserved activity and cytotoxicity, but GD2.CAR-γδ T cells rapidly acquire depletion status (368), requiring further modification. GD2.CAR-NKT cells have low persistence (369), but the inclusion of IL15 in the construct may address this problem and enhance tumor infiltration and antitumor activity *in vivo* (370). It was also observed that GD2.CAR-NKT cells did not contribute to the development of GvHD, whereas GD2.CAR T cells were lethal (369). In the GD2.CAR clinical trial, IL15-enhanced NKT cells showed safety and objective responses (371). CAR-NKT cells, compared to CAR-NK cells, were also shown to better regulate the immune system, infiltrate tissues, are resistant, and differ by memory phenotype (372).

Since TME is a major obstacle for CAR-T, arming M1 macrophages with CAR (CAR-M) was proposed as an alternative approach. Macrophages can penetrate the tumor much more easily, while having high phagocytic capacity, secreting proinflammatory cytokines and lytic molecules (ROS, iNOS, NO), presenting antigens, and interacting with immune cells (373). However, several challenges were encountered to realize this approach, particularly, *in vitro* and *in vivo* propagation and gene transfer into primary macrophages. GD2.CAR-M were derived from pluripotent stem cells using the CRISPR/Cas9 method and have potent cytotoxic activity *in vitro* and *in vivo* (374), which may serve as a platform for further testing.

## 6 Conclusion

Current research is aimed at improving the safety and efficacy of treatment. The success of antitumor therapy largely depends on a properly selected target antigen. GD2 expression is detected on the cell surface of a wide range of solid tumors at high levels. It is restricted to neoplasms and is not lost after treatment. Suitable agents for targeting GD2 are those capable of recognizing antigens of glycolipid origin, such as monoclonal antibodies or a chimeric antigen receptor. Preclinical and clinical studies showed that combination therapy was the most promising treatment compared to monotherapy, in particular, targeting not only tumor cells but also the microenvironment. In addition, the treatment should be safe, scalable, and cost-effective. From this point of view, the most promising area of genetic engineering is humanized monoclonal antibodies, which showed clinical efficacy and were officially approved. At the same time, cell therapy shows promising results. CAR cells have a direct cytotoxic effect on tumor cells, and various CAR modifications also make it possible to influence both TME and immunocompetent cells. In the future, optimization of new generations of CAR design and protocols for obtaining genetically modified cells should be aimed at improving safety and overcoming early cellular depletion. Such modifications may include replacing chimeric scFv with humanized scFv, reducing tonic signaling from the CAR receptor, using NK, NKT cells, or macrophages that do not induce GvHD reactions and pronounced toxic side effects, and optimizing protocols to produce scaled ready-to-use cells without functional signs of depletion.

## Author contributions

JP: Conceptualization, Visualization, Writing – original draft, Writing – review & editing. JS: Writing – review & editing. SS: Funding acquisition, Project administration, Resources, Supervision, Writing – review & editing.
